# HEARING IMPAIRMENT, COGNITIVE PERFORMANCE, AND BETA-AMYLOID DEPOSITION IN THE ARIC-PET AMYLOID IMAGING STUDY

**DOI:** 10.1093/geroni/igz038.2032

**Published:** 2019-11-08

**Authors:** Jennifer A Deal, Andreea Rawlings, A Richey Sharrett, Nicholas Reed, Joshua Betz, Thomas Mosley, Frank Lin, Rebecca Gottesman

**Affiliations:** 1 Johns Hopkins University Bloomberg School of Public Health, Baltimore, Maryland, United States; 2 Kaiser Permanente Center for Health Research, Portland, Oregon, United States; 3 Johns Hopkins University, Baltimore, Maryland, United States; 4 University of Mississippi Medical Center MIND Center, Jackson, Mississippi, United States

## Abstract

Hearing impairment is a risk factor for dementia but the mechanism underlying this association is unknown. We investigated the relationship between hearing and cognitive performance and brain β-amyloid deposition in 252 adults aged 67-88 years (37% black race) without dementia from three U.S. communities. Global cortical standardized uptake value ratios (SUVRs) were calculated from florbetapir-positron emission tomography scans, with elevated SUVR defined as >1.2 (the median value). Air conduction hearing threshold levels for the frequencies of 0.5, 1, 2 and 4 kHz were obtained by pure tone audiometry and averaged for the better-hearing ear to yield a pure tone average (PTA) in decibels hearing level (dB). A composite cognitive score was created from ten neuropsychological tests summarized using latent variable methods. Multivariable-adjusted linear and Poisson regression with robust standard errors were used to estimate the average difference in cognitive scores and prevalence of elevated SUVR, respectively, by hearing impairment status. In analyses adjusted for age, sex, education and APOE ε4 status, hearing was not associated with elevated SUVR [prevalence ratio per 10 db increase (worse hearing) = 0.94 (95% CI: 0.84, 1.04)]. Results did not differ by race. In contrast, each 10 db increase in hearing impairment was associated with 0.08 standard deviation (95% CI: 0.02, 0.15) lower cognitive score, after adjustment for demographic and cardiovascular factors. Poorer hearing is associated with poorer cognitive performance but not with amyloid deposition, suggesting that the mechanism underlying the relationship between hearing and cognition may be independent of Alzheimer’s-related pathologic brain changes.

